# Supplementary material for: Molecular basis of the autoregulatory mechanism of motor neuron-related splicing factor 30
Source: J Biol Chem. 2025 Jul 25;301(9):110522. doi: 10.1016/j.jbc.2025.110522 (PMC12398788; doi:10.1016/j.jbc.2025.110522)
Supplement: Supporting Figures and Tables [file mmc1.pdf]

Supporting information

Molecular basis of the autoregulatory mechanism of motor neuron-related splicing factor 30

Keiichi Izumikawa[1,#,*], Tatsuya Shida[1,#], Yuuka Onodera[1], Yuito Tashima[1], Sotaro Miyao[1,2], Tomomi Suda[3], Yasuyuki Suda[3], Ryosuke Kamimura[1], Maiko Nagai[1], Minoru Sugihara[4], Tamotsu Noguchi[5], Masami Nagahama[1,*]

[#] These authors contributed equally to this work.

***Correspondence**: Keiichi Izumikawa; k-izumikawa@my-pharm.ac.jp, Masami Nagahama; nagahama@my-pharm.ac.jp

Laboratory of Molecular and Cellular Biochemistry, Meiji Pharmaceutical University, 2-522-1, Noshio, Kiyose-shi, Tokyo, 204-8588, Japan. Tel: +81-042-495-8798

Supporting Experimental Procedure

Rescue assay

SPF30-mAID cells, cultured in 6-well plates, were transfected with 500 ng of either pcDNA5/FRT/TO SPF30 (no tag) or pcDNA5/FRT/TO SPF30(WT)-FLAG-6His (referred to as SPF30-FLAG) using FuGENE 4K (Promega, Madison, WI, USA) according to the manufacturer's instructions. As a control, pcDNA3.1(+) was used as an empty vector (EV) for both SPF30 (no tag) and SPF30-FH. Two hours after transfection, cells were treated with or without 0.25 µM 5-Ph-IAA. After 48 hours of transfection, cells were harvested in ice-cold PBS. Half of the cells were lysed using RIPA buffer (50 mM Tris–HCl pH 8.0, 150 mM NaCl, 1% IGEPAL CA-630, 0.1% SDS, 0.5% sodium deoxycholate) containing 1 mM PMSF for 10 min on ice. Lysates were centrifuged at 20,000 × *g* and 4°C for 10 min, and the supernatants were collected as total cell extracts for protein analysis. The remaining cells were subjected to RNA extraction and subsequent RT-qPCR analysis, as described in the "RT-qPCR and RT-PCR analysis" section of the Experimental procedures in the main manuscript.

Supporting Tables

Supplementary Table S1. List of antibodies used in this study.

Antibody	Host/isotype	Supplier	Product code	Used for
GAPDH (6C5)	Mouse / IgG1	Ambion	AM4300	WB
FLAG (M2)	Mouse / IgG1	SIGMA	F3165	WB
FLAG	Rabbit	SIGMA	F7425	ICS
SPF30 (SMNDC1)	Rabbit	INVITROGEN	PA5-31148	WB, ICS
SC-35	Mouse / IgG1	SIGMA	SAB4200725	ICS
Anti-mouse IgG, HRP-linked Antibody	Horse	Cell Signaling Technology	#7076	WB
Anti-Rabbit IgG (HTL)-HRP Conjugate	Goat	Bio-Rad	#1706515	WB
Anti-mouse IgG (HTL)-HRP Conjugate	Goat	Bio-Rad	#1706515	WB
Anti-mouse IgG (Fc BP)-HRP Conjugate		Santa Cruz Biotechnology	sc-525409	WB
Goat anti-Rabbit IgG (H+L) Cross-Adsorbed Secondary Antibody, Alexa Fluor 488	Goat	Themo Fisher Scientific	A-11008	ICS
Goat anti-Mouse IgG (H+L) Cross-Adsorbed Secondary Antibody, Alexa Fluor 594	Goat	Themo Fisher Scientific	A-11005	ICS

Supplementary Table S2. List of primers used in this study.

Primer Name	Sequence 5' --> 3'	Used for
KI-173	CATGTACGTTGCTATCCAGGC	ACTB-Fw, qPCR
KI-174	CTCCTTAATGTCACGCACGAT	ACTB-Rv, qPCR
KI-58	AACAAGATGAGATTGGCA	ACTB-Fw, RT-PCR
KI-59	GACCAAAAGCCTTCATACAT	ACTB-Rv, RT-PCR
KI-39	CTAGCAATTCATTGGAACACCA	SPF30 3'UTR(Exon 6)-Fw, qPCR
KI-40	GCCTGGGAAACAAGTTATTGAG	SPF30 3'UTR(Exon 6)-Rv, qPCR
KI-41	AGCGGAGATTGAGGAGATAGATG	SPF30 CDS(Ex4)-Fw, qPCR, RT-PCR
KI-42	GGTTCAACAGTGGAGTCACTTC	SPF30 CDS(Ex4)-Rv, qPCR, RT-PCR
KI-43	TTTCCTGCTACTGCTACTGCTG	SPF30 Ex1-Fw, RT-PCR
KI-44	CTGCAGAGATGAAATCCAACAG	SPF30 Ex6-Rv, RT-PCR
KI-78	TTCCTCCCTCAAGTCTGAGTTC	SPF30 Int2-Rv, qPCR
KI-79	TACAAAGCTCAGCTCCAGCA	SPF30 Ex2-Fw, qPCR
KI-97	ACACCTGTAAAGATATCATGGTGTC	SPF30 Ex4a-Rv, qPCR, RT-PCR
KI-130	GAACTAACCAAAGACCTTCTGTCAA	SPF30 Ex3-Fw, qPCR, RT-PCR for Ex3/NlucP
KI-423	TGAGGAGAAGTCTGCCGTTACT	HBB(18-)-Fw, RT-PCR for HBB/NlucP
KI-441	CACCCCGAGATTCTGAAACAAA	NlucP-Rv, RT-PCR for Ex3/NlucP or HBB/NlucP
KI-445	ACCTATTACACGAAGGACCTCC	UPF1-Fw, qPCR
KI-446	ACGTCCGTTGCAGAACCAC	UPF1-Rv, qPCR
KI-5	CGCAAATGGGCGGTAGGCGTG	Construction of pcDNA5FRT/TO SPF30(ΔC2, ΔC3)-FLAG-6His
KI-16	AGCTTGGTACCGAGCTCGACCGCCATGTCAGAGGATTTAGCAAAGCAG	Construction of pcDNA5FRT/TO SPF30(WT, ΔC1, no tag)-FLAG-6His<br>Construction of pcDNA5/FRT/TO SPF30(W83A)-FLAG-6His
KI-17	TCCTTGTAATCGGTACCGGCTTGAGGCATCAAATGCCTGACATT	Construction of pcDNA5FRT/TO SPF30(WT, ΔN)-FLAG-6His<br>Construction of pcDNA5/FRT/TO SPF30(W83A)-FLAG-6His
KI-101	AGCTTGGTACCGAGCTCGGATCCACCGCCATGTCTACTCAACCTACTCATTCATGG	Construction of pcDNA5FRT/TO SPF30(ΔN)-FLAG-6His
KI-99	TCCTTGTAATCGGTACCGGCTATTCTCTGAGCTTTTTTCAAAGC	Construction of pcDNA5FRT/TO SPF30(ΔC1)-FLAG-6His
KI-102	TATAGGATCCAGAAGCAAAACTGTCTGAACT	Construction of pcDNA5FRT/TO SPF30(ΔTudor)-FLAG-6His
KI-103	TATAGGATCCAAGGAGGACAGTGGCAACAAA	Construction of pcDNA5FRT/TO SPF30(ΔTudor)-FLAG-6His
KI-316	TATAggatccCACACTCTCAGGTGAAGCAAAAATA	Construction of pcDNA5/FRT/TO SPF30(ΔC4)-FLAG-6His,<br>Construction of pCold-I SPF30(ΔC4)
KI-319	tataggatccGCCGGTACCGATTACAAGGACGAC	Construction of pcDNA5/FRT/TO SPF30(ΔC4, ΔC5)-FLAG-6His
KI-318	TATAggatccAGCAATTCCACAGGTTCCTACTCC	Construction of pcDNA5/FRT/TO SPF30(ΔC5)-FLAG-6His,<br>Construction of pCold-I SPF30(ΔC5)
KI-392	TATAggtaccAGAATAGGCTCTGTTGTTGAATTG	Construction of pcDNA5/FRT/TO SPF30(ΔC2)-FLAG-6His
KI-393	TATAggtaccCCTCTTTACCTGGCCTTTTTTGTT	Construction of pcDNA5/FRT/TO SPF30(ΔC3)-FLAG-6His
KI-442	AGTGTGACTGGTAAAgctGGAGTAGGAACCTGT	Construction of pcDNA5/FRT/TO SPF30(FSV3A)-FLAG-6His,<br>Construction of pCold-I SPF30(FSV3A)
KI-443	ACAGGTTCCTACTCCagcTTTACCAGTCACACT	Construction of pcDNA5/FRT/TO SPF30(FSV3A)-FLAG-6His,<br>Construction of pCold-I SPF30(FSV3A)
KI-444	TATACTCGAGttattgaggcatcaaatgcctgac	Construction of pcDNA5FRT/TO SPF30(no tag)
KI-231	tataGGATCCAtacccttatgacgtcccggactac	Construction of pNanoLucP
KI-232	TATAgcggccgcTTATGACGTTGATGCGAGCTGAAG	Construction of pNanoLucP
KI-205	TATAaagcttACCGCCATGGAACTAACCAAAGACCTTCTGTCA	Construction of pNanoLucP SPF30(Ex3-Int3-Ex4)(WT)
KI-226	TATAggatccggGCCATAACCAGCAAAGGTGATTG	Construction of pNanoLucP SPF30(Ex3-Int3-Ex4)(WT)
KI-305	TGCTGGTGGTCGAGATTGAGGAGATAGATGAAGAA	Construction of pNanoLucP SPF30(Ex3-Int3-mEx4)(mut1)
KI-306	CGACCACCAGCAACCTGTAAAGATATCATGGCTGT	Construction of pNanoLucP SPF30(Ex3-Int3-mEx4)(mut1)
KI-255	TTCCCACCCTCAGGTGTTATGAAGCGGAGATTGAG	Construction of pNanoLucP SPF30(Ex3-Int3-mEx4a)(mut2)
KI-256	AGGGTGGGAAATGGCTGTAAGCAGGTGAGTAAAGG	Construction of pNanoLucP SPF30(Ex3-Int3-mEx4a)(mut2)
KI-257	TTCCCACCCTCAGCCATGATATCTTTACAGGTGTT	Construction of pNanoLucP SPF30(Ex3-mInt3-Ex4)(mut3)
KI-258	AGGGTGGGAAAGTAAAGGTTTAGTACTCAGCCTAC	Construction of pNanoLucP SPF30(Ex3-mInt3-Ex4)(mut3)
KI-251	tataAAGCTTACCATGGTGCATCTGACTCCTGAGG	Construction of pNanoLucP HBB(Ex1-Int1-Ex2)
KI-252	tataGGATCCTTAGGGTTGCCCATAACAGCATCA	Construction of pNanoLucP HBB(Ex1-Int1-Ex2)
KI-437	ACTCAGATCTCGAGCTCAAGCTTACCATG	Construction of pNanoLucP SPF30(HBB(Ex1-Int1)-Ex4)(mut4)<br>Construction of pNanoLucP SPF30(HBB(Ex1-Int1)-Ex4)(mut5)
KI-438	GGTGAGTAAAGGTTTAGTACTCAGAGTCAGTGCCTATCAGAAA	Construction of pNanoLucP SPF30(HBB(Ex1-Int1)-Ex4)(mut4)
KI-439	TAAGCAGGTGAGTAAAGGAGAGTCAGTGCCTATCAGAAA	Construction of pNanoLucP SPF30(HBB(Ex1-Int1)-Ex4)(mut5)
KI-143	tataGAGCTCATGTCAGAGGATTTAGCAAAGCAG	Construction of pCold-I SPF30(WT, ΔC1, ΔC2, ΔC3, ΔC4)
KI-125	TATATCTAGACTACTTGTCATCGTCGTCCTTGTA	Construction of pCold-I SPF30(WT)
KI-153	TATAggatccTATTCTCTGAGCTTTTTTCAAAGC	Construction of pCold-I SPF30(ΔC1)
KI-219	TATAggatccAGAATAGGCTCTGTTGTTGAATTG	Construction of pCold-I SPF30(ΔC2)
KI-220	TATAggatccCCTCTTTACCTGGCCTTTTTTGTT	Construction of pCold-I SPF30(ΔC3)
KI-320	GATATCAGTCGACGGATCCGGTACCGATTA	Construction of pCold-I SPF30(ΔC5)
KI-370	CCTCTTTACCTGGCCTTTTTTGTT	Construction of pCold-I SPF30(ΔC6)
KI-371	ACTGGTAAAGTTGGAGTAGGAACC	Construction of pCold-I SPF30(ΔC6)
KI-178	CCACTACCACTGGGCTCATT	Library preparation for deep sequencing, SPF30-Exon1-Fw
KI-179	CGGTGCCATTTTCTTCATCT	Library preparation for deep sequencing, SPF30-Exon4-Rv
SM-1	CACCAAAGTTGGAGTAGGAACCTG	Construction of peSpCas9(1.1)-2×sgRNA SPF30
SM-2	AAACCAGGTTCCTACTCCAACTTT	Construction of peSpCas9(1.1)-2×sgRNA SPF30
SM-3	ACTGGAGCTCACAGGCCCCACAGTAAACCACA	Construction of pBlueScript II SK (+)-SPF30-HA
SM-4	AGCTGGTACCGCAGGTTGGTGTTTTTGCCTTGG	Construction of pBlueScript II SK (+)-SPF30-HA
SM-5	GGATCCTAAAGGGCTTTACATTTACC	Construction of pBlueScript II SK (+)-SPF30-HA
SM-6	TTGAGGCATCAAATGCCTGACATTG	Construction of pBlueScript II SK (+)-SPF30-HA
SM-7	AAGGTAGGTGTTGGAACCTGTGGAATTGCTG	Construction of pBlueScript II SK (+)-SPF30-HA
SM-8	ACCAGTCACACTCTCAGGTGAAGC	Construction of pBlueScript II SK (+)-SPF30-HA
IS-1	GCGAGTGAAGATGGACAGTGTTATG	Construction of pcDNA5/FRT/TO SPF30(W83A)-FLAG-6His
IS-2	GACTGCCATACACTTGTCTCCTAC	Construction of pcDNA5/FRT/TO SPF30(W83A)-FLAG-6His
YO-1	AAAAAATCGATATGTCAGAGGATTTAGCAAAGCAGC	Construction of pcDNA5/FRT/TO SPF30(W83A)-FLAG-6His
YO-2	AAAGATATCAATTGAGGCATCAAATGCCTGACATTG	Construction of pcDNA5/FRT/TO SPF30(W83A)-FLAG-6His

siRNA	Sequence 5' --> 3'	Used for
scRNA	SIC-002; Universal Negative Control #2	Negative Control to siUPF1
si UPF1-sense	GAGAUAUGCCUGCGGUACA[dT][dT]	UPF1(siRNA), SASI_Hs01_00101017/UPF1
si UPF1-antisense	UGUACCGCAGGCAUAUCUC[dT][dT]	UPF1(siRNA), SASI_Hs01_00101017_AS/UPF1

Supplementary Table S3. List of plasmids used in this study.

Plasmid	Methods for construction
pcDNA5/FRT/TO DAP-Ex2-Int2-Ex3	Izumikawa et al. 2018
pcDNA5FRT/TO FLAG-Chtop	Izumikawa et al. 2016
pOG44	Themo Fisher Scientific (USA)
Modified pBICEP-CMV2 SPF30-FLAG	Ishida et al. 2021
Modified pBICEP-CMV2 SPF30(W83A)-FLAG	
pEBG SPF30(WT)	Ishida et al. 2021
pEBG SPF30(W83A)	
pcDNA5FRT/TO SPF30(no tag)	
pcDNA5FRT/TO SPF30(WT)-FLAG-6His	
pcDNA5FRT/TO SPF30(ΔN)-FLAG-6His	
pcDNA5FRT/TO SPF30(ΔTudor)-FLAG-6His	
pcDNA5FRT/TO SPF30(ΔC1)-FLAG-6His	
pcDNA5FRT/TO SPF30(ΔC2)-FLAG-6His	
pcDNA5FRT/TO SPF30(ΔC3)-FLAG-6His	
pcDNA5FRT/TO SPF30(ΔC4)-FLAG-6His	
pcDNA5FRT/TO SPF30(ΔC5)-FLAG-6His	
pcDNA5FRT/TO SPF30(W83A)-FLAG-6His	
pcDNA5FRT/TO SPF30(FSV3A)-FLAG-6His	
pCold-I SPF30(WT)	
pCold-I SPF30(ΔC1)	
pCold-I SPF30(ΔC2)	
pCold-I SPF30(ΔC3)	
pCold-I SPF30(ΔC4)	
pCold-I SPF30(ΔC5)	
pCold-I SPF30(ΔC6)	
pCold-I SPF30(FSV3A)	
pNlucP SPF30(Ex3-Int3-Ex4)(WT)	
pNlucP SPF30(Ex3-Int3-mEx4)(mut1)	
pNlucP SPF30(Ex3-Int3-mEx4a)(mut2)	
pNlucP SPF30(Ex3-mInt3-Ex4)(mut3)	
pNlucP SPF30(HBB(Ex1-Int1)-Ex4)(mut4)	
pNlucP SPF30(HBB(Ex1-Int1)-Ex4)(mut5)	
pNlucP HBB(Ex1-Int1-Ex2)	
peSpCas9(1.1)-2×sgRNA	Addgene#80768
peSpCas9(1.1)-2×sgRNA SPF30	
pBlueScript II SK (+)	-
pBlueScript II SK (+) SPF30-HA	
pMK293	RIKEN DNA BANK
pMK393	RIKEN DNA BANK
pBlueScript II SK (+) SPF30-HA-pMK293	
pBlueScript II SK (+) SPF30-HA-pMK393	
pcDNA3.1(+)	

Supporting Figures

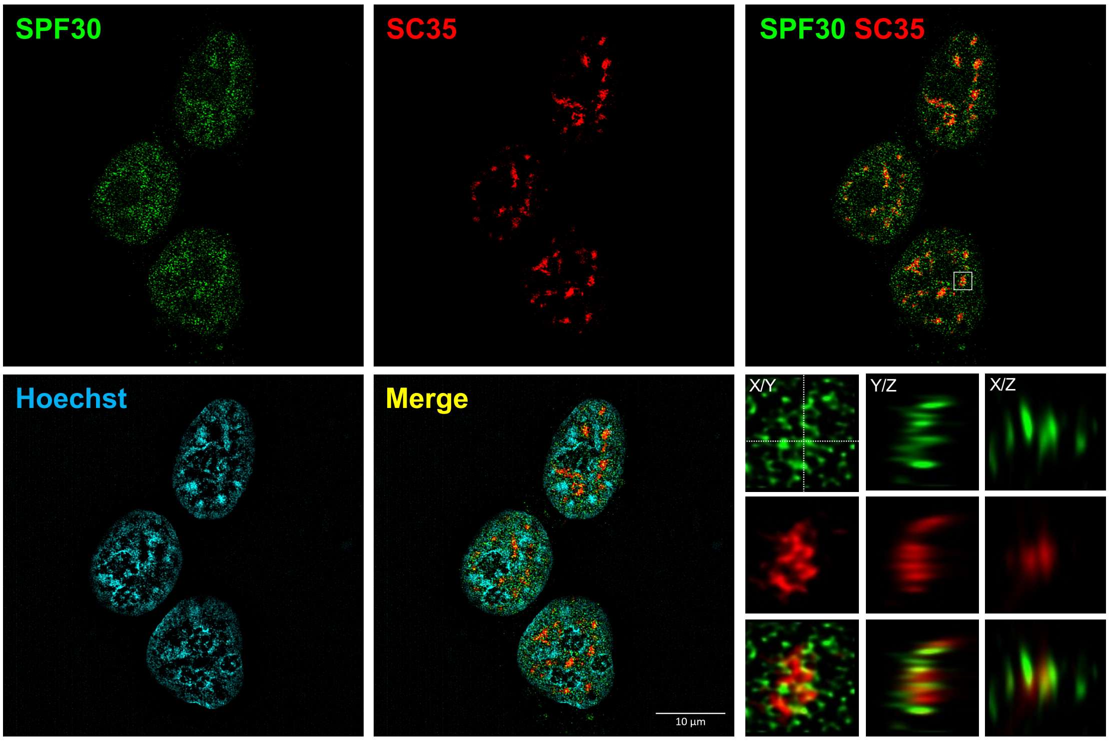

Supplementary Figure S1.

Lattice SIM^2 visualization of immunocytostaining of SPF30 (green), SC35 (red), Hoechst33342 (blue). In 293FTR cells, SPF30 and SC35 were stained with anti-SPF30 (rabbit IgG), and anti-SC35 (mouse IgG) antibodies as the first antibodies, and stained with Alexa488-conjugated anti-rabbit IgG and Alexa594-conjugated anti-mouse IgG as the secondary antibodies. SC35 was used as nuclear speckles marker. Hoechst33342 (Hoechst) was used as nuclear marker. Scale bar; 10 μm. Cross-sectional images along the x/z and y/z planes are shown at the positions marked by white dotted lines along the X- and Y-axes (Bottom right 9 panels).

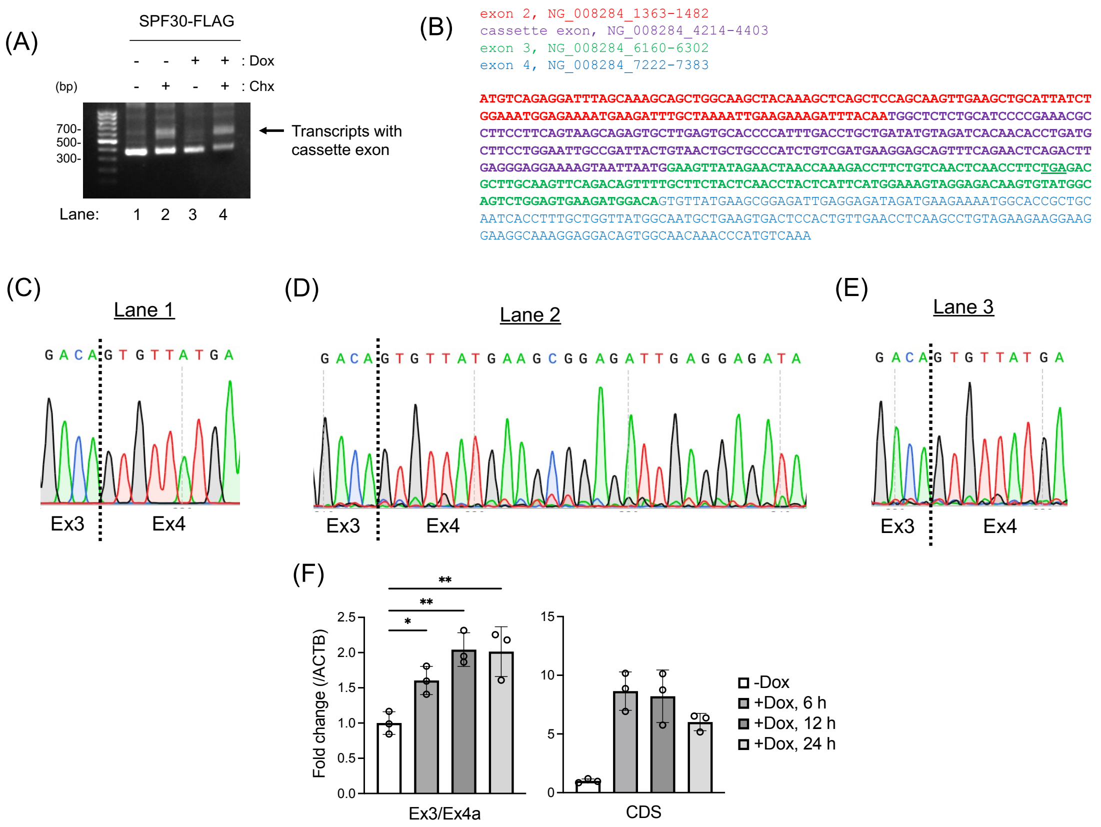

Supplementary Figure S2.

SPF30 transcripts with the cassette exon within intron 2 and exon 4a were generated upon CHX or SPF30 overexpression. (A) RT-PCR analysis of SPF30 transcripts for the deep sequencing. SPF30-FLAG–inducible cells were treated with/without Dox for 48 h and CHX for 4 h, and total RNA was extracted. Endogenous SPF30 transcripts were detected by RT-PCR using exon 1 and exon 4 primer sets (KI-178/KI-179). (B) Sequence of SPF30 transcripts with the cassette exon, from exon2 to exon4, was annotated by the deep sequencing. (C–E) The PCR fragments with 500 bp, amplified by Ex1/Ex4, in lane 1 (C), lane 2 (D), and lane 3 (E) of Figure 2B were annotated by Sanger sequencing. Wave data at the junction of exon 3 and exon 4 from each Sanger sequencing were presented. (F) SPF30-FLAG–inducible cells were treated with Dox for 0, 6, 12, and 24 h, and SPF30 transcripts (Ex3/Ex4a) present in exon 4a were measured by RT-qPCR analysis. Induction of SPF30-FLAG (SPF30 CDS) was confirmed using a CDS primer set. Data are presented as the mean ± standard deviation (SD) of values from three independent experiments. *P < 0.05, **P < 0.01 (Dunnett's test).

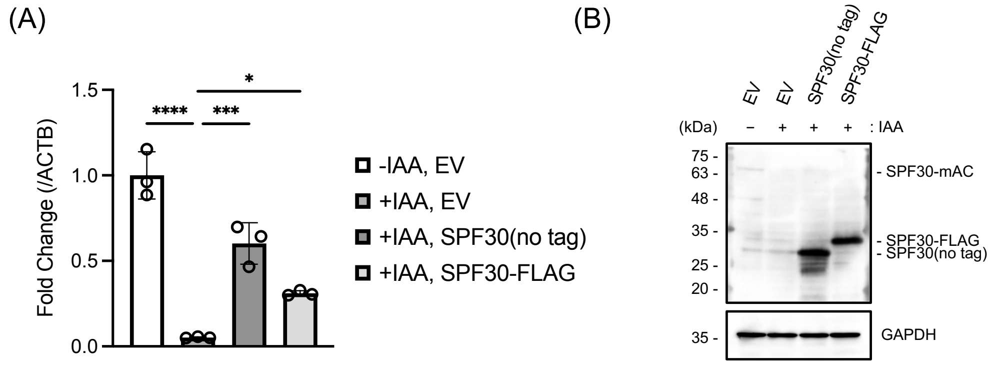

Supplementary Figure S3.

Rescue assays for the reduced expression of SPF30 transcripts containing exon 4a following SPF30 knockdown. (A) SPF30-mAID cells were transfected with an empty vector (pcDNA3.1), a tag-less SPF30 expression vector (SPF30(no tag)), or an SPF30-FLAG-His expression vector (SPF30-FLAG). After 2 h of transfection, cells were treated with or without (+IAA or –IAA, respectively) for 48 h. The relative expression levels of SPF30 transcripts containing exon 4a (Ex3/Ex4a) were measured by RT-qPCR analysis. Data are presented as the mean ± SD of values from three independent experiments. *P < 0.05, ***P < 0.001, ****P < 0.0001 (Dunnett's test vs. +IAA, EV). (B) Expression levels of endogenous SPF30 (SPF30-mAID-mCherry; SPF30-mAC), and transiently expressed SPF30 (untagged or FLAG-tagged), as shown in the experiments in (A), were analyzed by western blotting using the anti-SPF30 antibody. GAPDH was shown as the loading control.

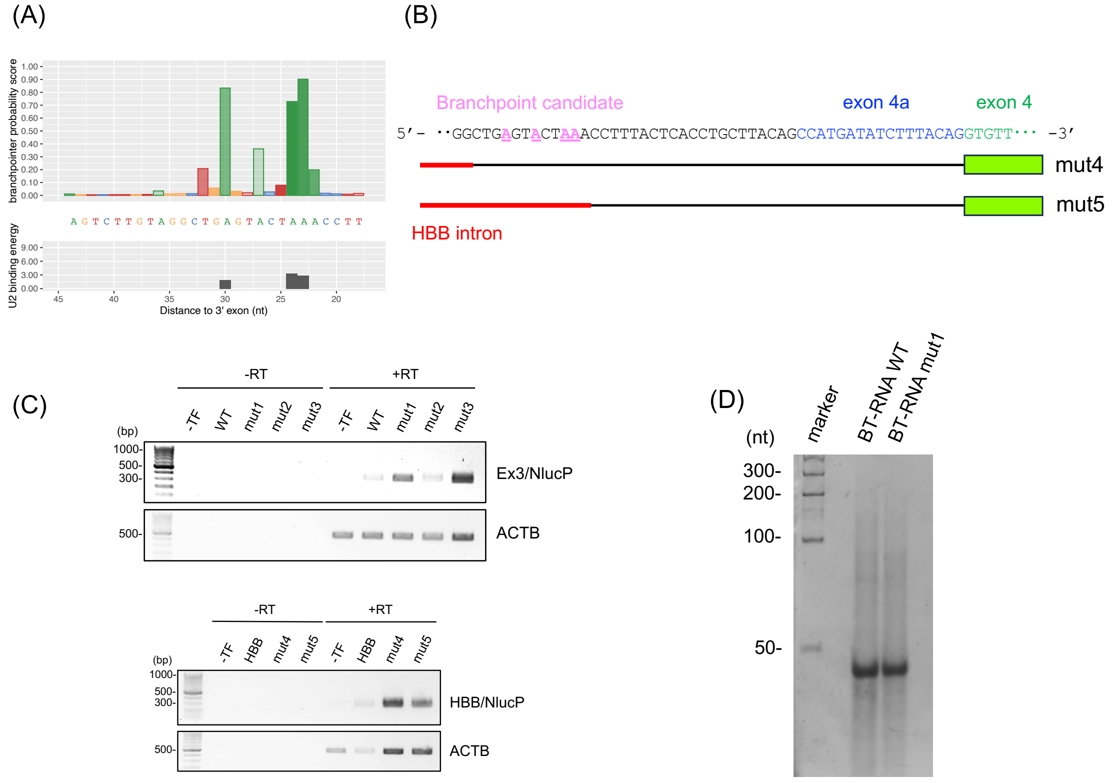

Supplementary Figure S4.

(A) Representation of the branchpoint candidates within intron 3 of SPF30 with branchpointer probability score predicted by the branchpointer software. (B) Schematic representation of NlucP mut4 and mut5. The predicted branchpoint candidates (magenta), the exon 4a–specific region (blue), and the canonical exon 4 (green) are shown in color. The DNA region of the HBB gene is shown in red. (C) RT-PCR analysis of transcripts derived from NlucP expression plasmids. Each NlucP expression plasmid was transfected into SPF30-mAID cells, and transcripts were detected via RT-PCR using primer sets targeting Ex3/NlucP or HBB/NlucP. ACTB was used as the loading control. (D) The biotinylated synthetic RNAs (BT-RNA WT and BT-RNA mut1) used in *in vitro* binding assays was separated by a denaturing urea-PAGE, and stained with SYBR gold. 1 pmol of RNAs was loaded to the denaturing urea-PAGE.

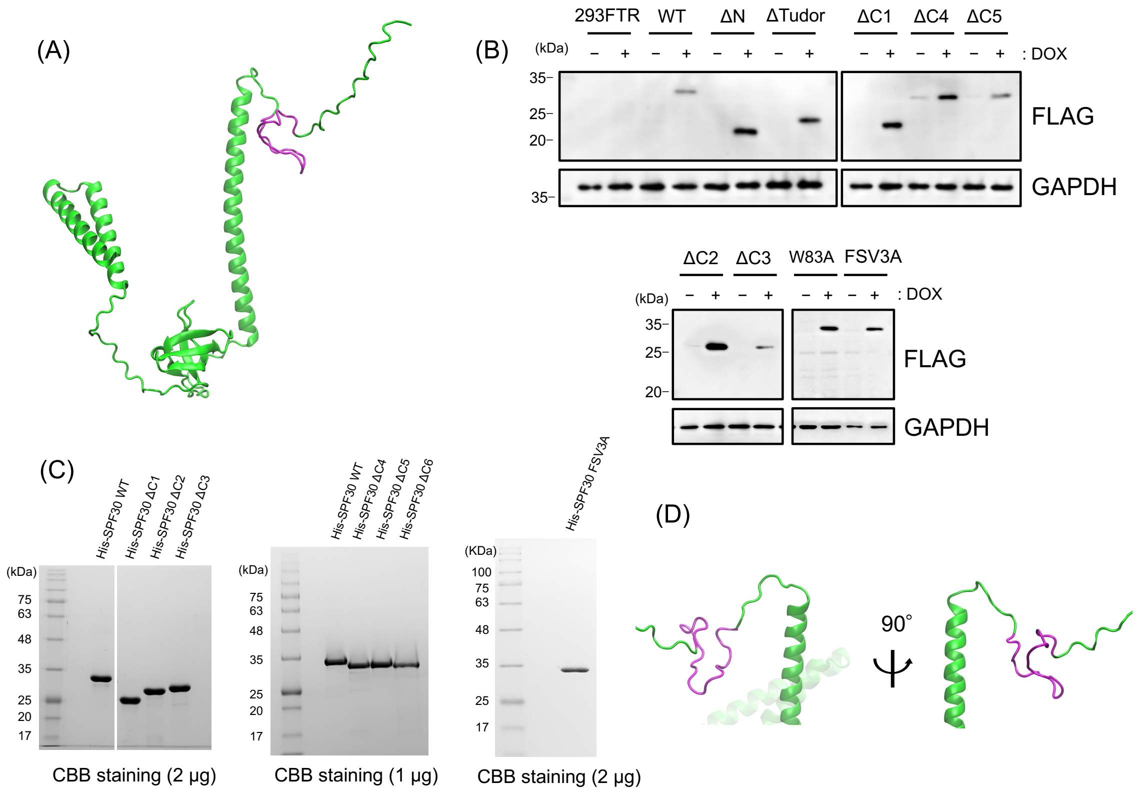

Supplementary Figure S5.

A structure model of SPF30 by Alphafold2. (A) A structure model of wild-type SPF30 were predicted by Alphafold2. A kink-like structure of SPF30 in the C-terminal intrinsically disordered region is colored magenta. (B) 293FTR cell lines capable of inducibly expressing mutants (WT, ΔN, ΔTudor, ΔC1, ΔC2, ΔC3, ΔC4, and ΔC5, W83A, and FSV3A) of SPF30 were treated without (–) or with (+) Dox for 24 h, and FLAG-tagged SPF30 were detected using anti-FLAG antibody, respectively. GAPDH was shown as loading control. 293FTR cell line was used as a parental cell. (C) The recombinants of 6xHis-tagged SPF30 mutants (ΔC1–ΔC6, FSV3A) were purified using Ni-NTA agarose from E. coli strain, and confirmed by SDS-PAGE and CBB stanning. Indicated amounts of proteins were loaded to SDS-PAGE. (D) The structure model of C-terminal α-helix and intrinsically disordered region is shown. A kink-like structure of SPF30 in the C-terminal intrinsically disordered region is colored magenta.

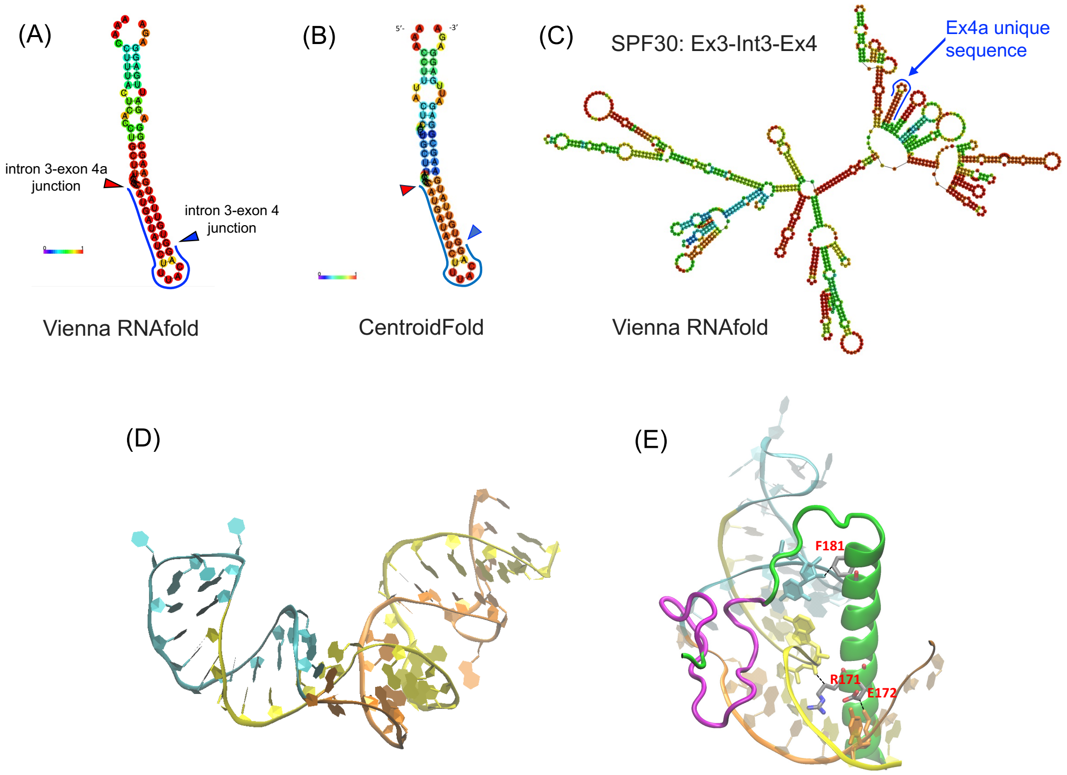

Supplementary Figure S6.

Secondary structure model of the SPF30 transcript containing exon 4a, as predicted by Vienna RNAfold (A) and CentroidFold (B). (A, B) The junction between intron 3 and exon 4a is indicated by a red arrowhead, and that between intron 3 and canonical exon 4 is indicated by a blue arrowhead. The unique 17 base sequence of exon 4a is shown as a blue line. (C) Secondary structure model of the SPF30 transcript spanning exon 3 to exon 4, predicted using Vienna RNAfold. (D) Based on the secondary structure shown in (A), a tertiary structure model was generated using RNAComposer (Automated RNA 3D structure modeling server). Exon 4 is shown in yellow, the unique sequence of exon 4a in cyan, and intron 3 in orange. (E) View of the C-terminal α-helix of SPF30 bound to RNA.

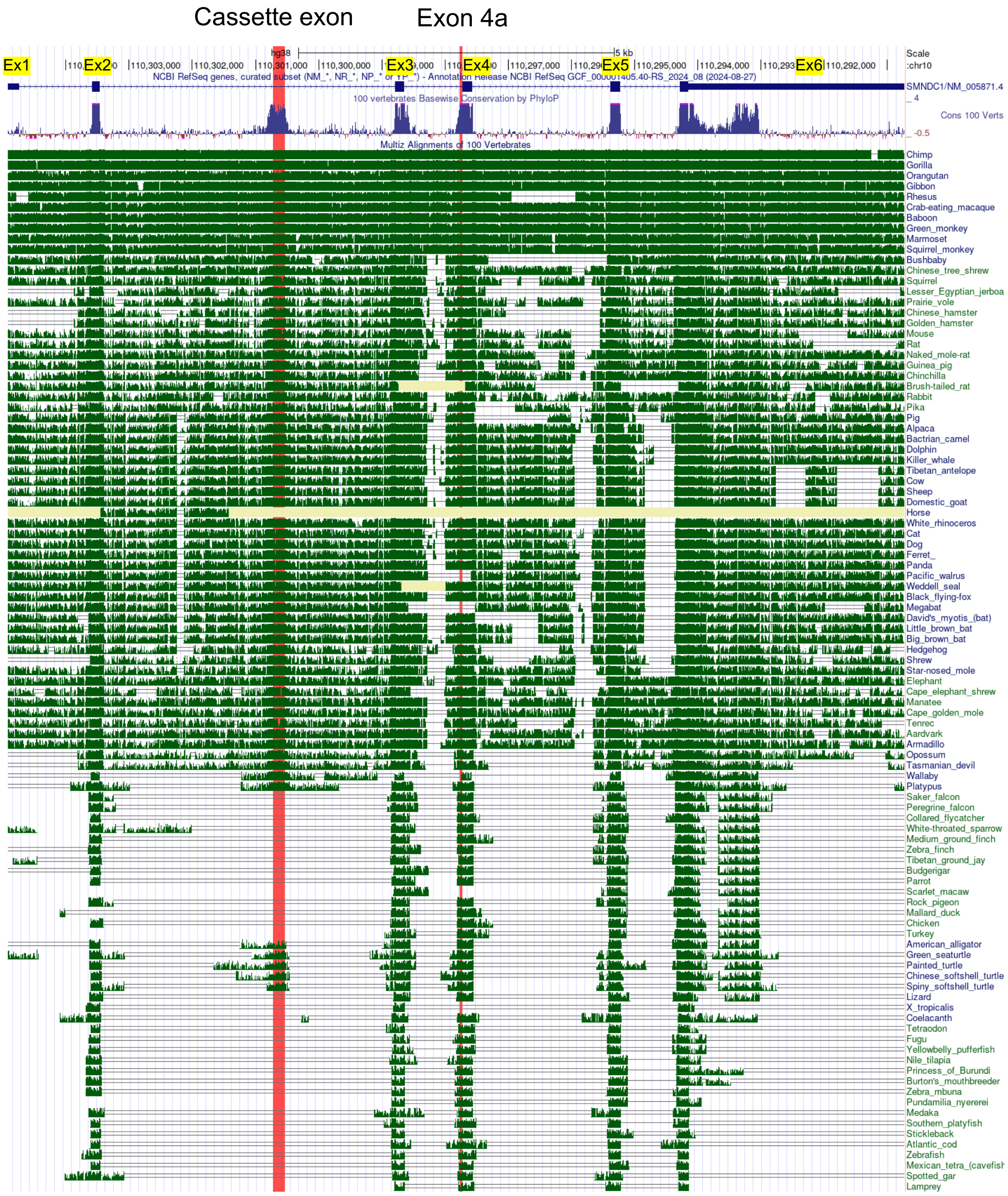

Supplementary Figure S7.

Comparison of conserved sequence of SPF30 gene (*SMNDC1*) among vertebrate species. The positions of exon 4a and the cassette exon within intron 2 are shown red lines. As a reference, human *SMNDC1* mRNA and DNA (NM_005871 and GRCh38/hg38, ch10) is used.

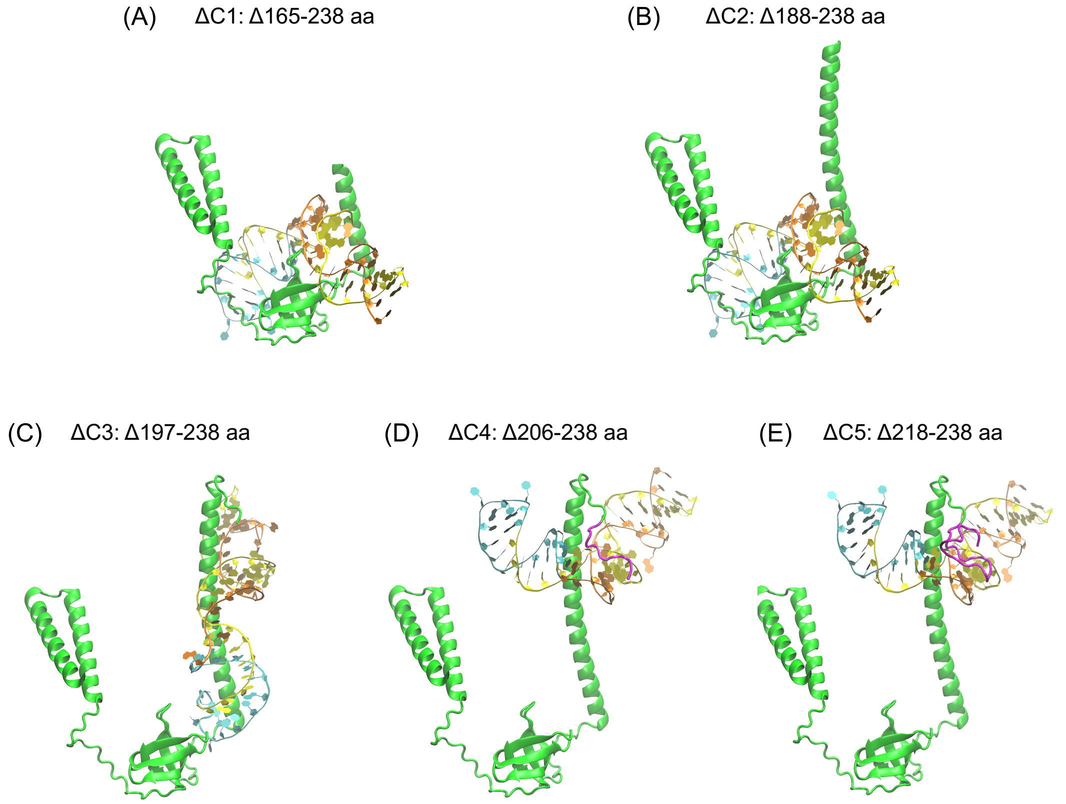

Supplementary Figure S8.

Docking models of SPF30 deletion mutants (A; ΔC1, B; ΔC2, C; ΔC3, D; ΔC4, E; ΔC5) and SPF30 transcripts containing exon 4a were predicted by HDOCK software. The kink-like structure of SPF30 is colored magenta. Exon 4 is colored yellow, the unique sequence of exon 4a is cyan, and intron 3 is orange.
